# Advanced analysis of free visual exploration patterns in schizophrenia

**DOI:** 10.3389/fpsyg.2013.00737

**Published:** 2013-10-11

**Authors:** Andreas Sprenger, Monique Friedrich, Matthias Nagel, Christiane S. Schmidt, Steffen Moritz, Rebekka Lencer

**Affiliations:** ^1^Department of Neurology, University of LuebeckLuebeck, Germany; ^2^Department of Psychiatry and Psychotherapy, University of LuebeckLuebeck, Germany; ^3^Asklepios Klinik Nord – Wandsbek, Clinic of Psychiatry and PsychotherapyHamburg, Germany; ^4^Department of Psychiatry and Psychotherapy, University Medical Centre Hamburg-EppendorfHamburg, Germany; ^5^Department of Psychiatry and Psychotherapy, University of MuensterMuenster, Germany

**Keywords:** schizophrenia, visual scanpath, visual exploration, focal processing, exploration strategy, attentional landscape, scanpath similarity

## Abstract

**Background:** Visual scanpath analyses provide important information about attention allocation and attention shifting during visual exploration of social situations. This study investigated whether patients with schizophrenia simply show restricted free visual exploration behavior reflected by reduced saccade frequency and increased fixation duration or whether patients use qualitatively different exploration strategies than healthy controls.

**Methods:** Scanpaths of 32 patients with schizophrenia and age-matched 33 healthy controls were assessed while participants freely explored six photos of daily life situations (20 s/photo) evaluated for cognitive complexity and emotional strain. Using fixation and saccade parameters, we compared temporal changes in exploration behavior, cluster analyses, attentional landscapes, and analyses of scanpath similarities between both groups.

**Results:** We found fewer fixation clusters, longer fixation durations within a cluster, fewer changes between clusters, and a greater increase of fixation duration over time in patients compared to controls. Scanpath patterns and attentional landscapes in patients also differed significantly from those of controls. Generally, cognitive complexity and emotional strain had significant effects on visual exploration behavior. This effect was similar in both groups as were physical properties of fixation locations.

**Conclusions:** Longer attention allocation to a given feature in a scene and less attention shifts in patients suggest a more focal processing mode compared to a more ambient exploration strategy in controls. These visual exploration alterations were present in patients independently of cognitive complexity, emotional strain or physical properties of visual cues implying that they represent a rather general deficit. Despite this impairment, patients were able to adapt their scanning behavior to changes in cognitive complexity and emotional strain similar to controls.

## Introduction

Deficits in the perception of social situations are suggested to underlie impaired social interaction in patients with schizophrenia (Addington et al., 2006). Reduced integration of visual context information in real-world situations has been suggested as one factor contributing to altered visual perception in patients (Green et al., 2005, 2008; Butler et al., 2008). Analyses of visual scanpaths provide important knowledge about how and when visual information is processed during visual exploration. A visual scanpath constitutes a sequence of voluntary saccades each shifting the focus of attention from one location of interest to the next thereby tracing the direction and extent of gaze when a subject extracts information from complex visual scenes (Noton and Stark, 1971). Scanpaths are affected both by sensory information such as physical stimulus properties, e.g., luminance or chromaticity contrasts, as well as the semantic relevance of a stimulus, e.g., cognitive complexity or emotional content (Bradley et al., 2011).

Previous studies using visual scanpath analyses in schizophrenia have mainly investigated face recognition (Frith et al., 1983; Gordon et al., 1992; Phillips and David, 1997; Streit et al., 1997; Williams et al., 1999) and the exploration of more complex scenes (Gaebel et al., 1987; Phillips et al., 2000). Some analyses focused on comparisons of quantitative eye-movement parameters, e.g., saccade frequency and amplitude (Phillips and David, 1997; Streit et al., 1997; Benson et al., 2007). Such studies have revealed restricted visual scanpaths in patients compared to healthy controls in terms of fewer and smaller saccades (Phillips and David, 1997, 1998; Williams et al., 1999; Loughland et al., 2002a). However, these kind of analyses do not provide information about whether patients and healthy individuals explore similar features in a scene with respect to their semantic and emotional contents or their physical properties. More recent studies suggested that patients tend to make fewer fixations on salient features in faces (eyes, nose, and mouth) than healthy controls (Williams et al., 1999; Loughland et al., 2002a,b). This pattern of visual avoidance was associated with deficits in the recognition of particular emotions (Loughland et al., 2002a; Green et al., 2003) and underlines the assumption that social perception in schizophrenia may be controlled by early restriction of input to visual cortex (Bestelmeyer et al., 2006; Green et al., 2008). Others have pointed out that during visual exploration visual information is gathered to test a-priori hypotheses and beliefs about the world (Friston et al., 2012). This model is based on the assumption that biological systems maximize the Bayesian evidence for their model of the world through an active sampling of sensory information (Friston et al., 2012). With respect to schizophrenia this suggests that scanpath abnormalities in patients reflect attention allocation and shifting driven by aberrant or more uncertain beliefs about the world compared to that of healthy individuals (Fletcher and Frith, 2009).

In contrast to this assumption, abnormalities of saccade frequencies in patients have also been reported from studies using abstract (Manor et al., 1999; Obayashi et al., 2003) and geometric stimuli (Kojima et al., 1989, 1990, 1992) which differed in complexity but were free of emotional content. Minassian et al. (2005) therefore suggested a general impairment of visual scanning and exploration independent of the semantic content of an image that may be regarded as a stable behavioral marker associated with schizophrenia (Bestelmeyer et al., 2006; Benson et al., 2012).

In the present study we were interested in spatial and temporal aspects of visual exploration behavior of social daily life scenes in patients with schizophrenia. Stimuli were taken from the Integrated Psychological Training (IPT) program for patients with chronic schizophrenia (Roder et al., 1997). One part of the IPT focuses on remediation of social perception by improving visual exploration strategies, training patients to systematically collect visual information from a given visual scene prior to drawing conclusions and interpreting its content. We used advanced scanpath analyses including examination of temporal changes in exploration behavior, cluster analyses, comparisons of attentional landscapes (Pomplun et al., 1996) and scanpath similarities. We also studied whether the stimuli’s cognitive complexity and emotional strain had different effects on exploration strategies in patients and controls and whether physical properties of fixation locations differed between groups.

## Materials and methods

### Subjects

Thirty-two psychopathologically stable patients from in- and out-services from two sites, the University Hospital of Luebeck (13 males; 2 females) and the University Hospital of Hamburg (9 males; 8 females) met DSM-IV-criteria for schizophrenia (American Psychiatric Association, 1994; Table 1). Patients did not differ between sites for age [*t*_(30)_ = 0.204, *p* = 0.232], mean illness duration [*t*_(30)_ = −0.59, *p* = 0.56] or mean age at onset of first psychotic symptoms [*t*_(30)_ = 0.80, *p* = 0.43]. Diagnoses were established using the German version of the Mini-International Neuropsychiatric Interview (M.I.N.I., Sheehan et al., 1998). Symptoms were assessed on the Positive and Negative Syndrome Scale (PANSS, Kay et al., 1987). Patients showed mild positive syndromes (~20th percentile), mild negative syndromes (~15th percentile) and a mild general psychopathology (~25nd percentile). The mean PANSS difference score of −1.45 (*SD* = 5.97) indicates a predominance of negative symptoms in patients.

**Table 1:** **Sociodemographic and illness related characteristics of the patient sample (given are means with standard deviation)**.

	**Patients total (*N* = 32)**
Age (years)	36.78 (12.42)
Illness duration (years)	8.38 (8.14)
Age at onset (years)	28.41 (9.18)
PANSS positive symptoms	12.87 (3.84)
PANSS negative symptoms	14.29 (5.75)
PANSS difference score	−1.45 (5.97)
PANSS general psychopathology	30.03 (7.02)

All patients were on medication, usually antipsychotics (olanzapine *N* = 6, quetiapine *N* = 5, amisulpride *N* = 4, risperidone *N* = 3, clozapine *N* = 2, ziprasidone *N* = 2, phenothiazine *N* = 2, flupentixole *N* = 1). Some patients additionally received antidepressants (venlafaxine *N* = 2, citaloprame *N* = 2, paroxetine *N* = 1, sertraline *N* = 1, trimipamine *N* = 1). All patients were off benzodiazepines for at least 48 h prior to testing.

Thirty-three control subjects (20 males, 13 females) with no reported history of a major psychiatric disorder were recruited to match for age with the patient group [range 24–61 years, mean age in Luebeck (*N* = 16): 35.1 years ±9.9; Hamburg (*N* = 17): 36.0 years ±11.0; *F*_group(1, 61)_ = 1.0, *p* = 0.66, *F*_site(1, 61)_ = 0.00, *p* = 0.99, no interaction of GROUP × SITE]. Inclusion criteria for all participants comprised: (1) age 18–65 years, (2) normal or corrected-to-normal vision, (3) no current or reported substance dependency and no substance abuse during 4 weeks prior testing, (4) no known systemic or neurological disease. Visual acuity and color vision were assessed using Landolt Ring charts and the Ishihara Color test. Each participant gave written informed consent after having carefully been informed about the study. The study was approved by the local ethics committees of the universities of Luebeck and Hamburg.

### Stimuli

**Figure 1:**
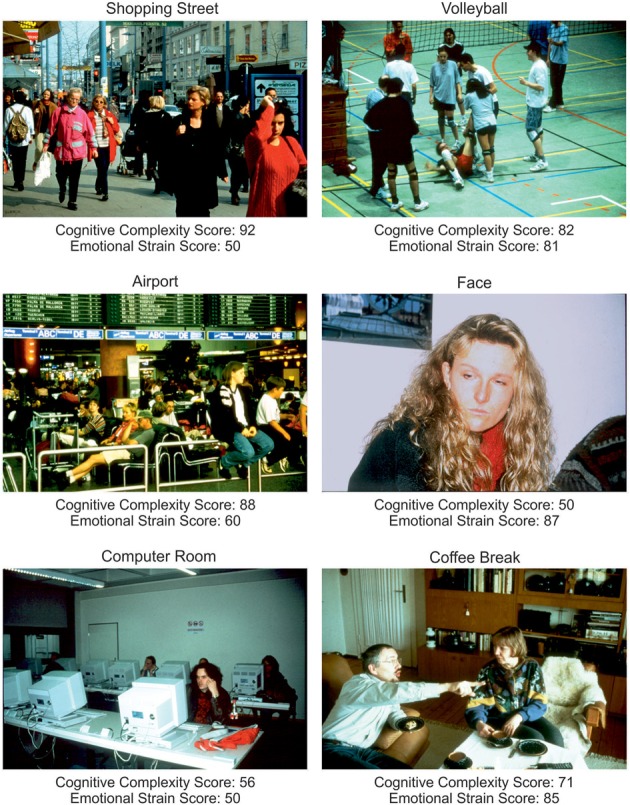
**Stimuli showing social daily life scenes (Roder et al., 1997).** Indicated are scores for cognitive complexity and emotional strain derived from the evaluations provided by Roder and co-workers. Higher scores indicate a higher cognitive complexity resp. emotional strain (see Materials and Methods).

Six color photos depicting social daily life scenes were chosen from the “Integrated Psychological Therapy Program for Patients with Schizophrenia” (Roder et al., 1997). Photos were selected according to their ratings on the two dimensions “cognitive complexity” and “emotional strain” provided by Roder et al. (1997). Complexity and emotional strain scores for each photo were calculated by multiplying the percentage values of each of the evaluation categories reported by Roder et al. (1997) with a weighting factor (low = 1, moderate = 2, high = 3), summing these up and dividing the sum by 3, Figure 1.

Photos were presented in randomized order for 20 s each and subjects were instructed to look at the photos as if they were spectators of the depicted scene. After each presentation, participants were asked to rate their emotional reaction to the photos on a 3-item rating scale (“none,” “moderate,” “strong”) by clicking a button on a keypad.

1. How much tension did you experience regarding the photo?
2. How much fear did you experience regarding the photo?
3. How much sadness did you experience regarding the photo?
4. How much joy did you experience regarding the photo?
5. How much aggression did you experience regarding the photo?

Additionally, participants were asked whether they felt they had enough time to look at the photo and whether they had been able to grasp the photo’s content on a 3 point scale (“hardly,” “partly,” or “completely”).

### Recording procedure

At both sites, assessment took place in a dimly lit room. In Luebeck, participants were seated 180 cm in front of a 151 × 120 cm screen, which equates to an angle of 45.3 × 36.9°. In Hamburg, participants were seated 57 cm in front of a 36.3 × 27 cm CRT monitor (19 inch) comprising a visual angle of 35.3 × 26.6°. Stimulus resolution was 1024 × 768 pixels at both sites. Subjects were instructed not to move their head during the recording. Eye and head movement recordings at both sites were assessed with the same device using a video-based EyeLink I eye tracking system (250 Hz, SR Research Ltd., Ottawa, ON, Canada). An additional camera tracked four infrared markers mounted on the visual stimulus display for head motion compensation and true gaze position tracking.

### Data analysis

A semi-automatic computer program written in MatLab® (R2010a, The Mathworks Inc., Natic, MA, USA) was used to read and calibrate the eye tracking data. Eye position data was filtered by a 100 Hz Gaussian filter. Saccadic eye movements were detected by identifying an initial eye velocity above 30°/s with its peak velocity occurring within a time window of 60 ms. Beginning and end of a saccade were defined as the points at which velocity crossed 20°/s with a minimum saccade amplitude of 0.3°. All saccades, blinks and artifacts were detected and checked manually. Saccades with amplitudes <0.6° were classified as corrective saccades and excluded from amplitude analyses since they reflect the relocation of an object of interest rather than a complete attentional shift to a new location. The following parameters were determined for each photo: location, number and duration of fixations, number and amplitudes of saccades and the resulting scanpath lengths as the sum of saccade amplitudes during the 20 s scanning time. Additionally, the exploration time of 20 s was divided into four intervals of 5 s each to test for changes over the exploration time.

### Cluster analyses

To investigate whether possible disturbances of fixation and saccade behavior led to alterations of areas of interest in patients compared to controls, fixation locations were used in N-2 cluster analyses. Euclidian distances of all fixations per photo were calculated and linkage parameters were obtained using Ward’s method (Bortz, 1999). Cluster and distance matrices were calculated for cluster solutions starting at 2 clusters up to N-1 clusters with *N* = number of fixations per photo. This method allows calculating distances within and between clusters for each cluster solution. The minimum of the ratio of distances within/between clusters was considered as the optimal cluster solution. The following parameters were obtained for each photo: total number of clusters, total number of fixations per cluster, total fixation time within each cluster (ms), and the total number of changes between clusters.

### Attentional landscapes

To examine the “where” question, thus, whether patients allocated attention to different locations than controls, fixations on photos of each subject were weighted by their duration and smoothed with a 2D Gaussian function with σ = 1° visual angle. Resulting maps are known as “attentional landscapes” (Pomplun et al., 1996). These maps were compared between healthy controls and patients using *T*-tests, Figure 5. To correct for multiple comparisons, the significance threshold was set to *p* < 0.001.

### Similarities between scanpaths

Fixation sequences of each subject were compared to each other subject in the combined control-patient group using the Needleman–Wunsch algorithm (Needleman and Wunsch, 1970) implemented in a modified version of the ScanMatch toolbox (Cristino et al., 2010). Photos were split into areas of a 12 × 8 grid and fixations were assigned to these grid areas. Sub matrices were calculated using the twofold standard deviation of the mean saccade amplitude per photo divided by grid size. Similarity scores for each subject compared to each of the 64 other subjects were determined resulting in similarity matrices of 65 × 65 similarity scores for each photo. Subsequently, median similarity scores in each subject were defined for their scanpath similarity with that of the control group and that of the patient group, for each photo. Thereafter, difference scores between these two similarity scores were calculated for each subject and photo. Positive values indicate a stronger scanpath similarity to the control group whereas negative values indicate a stronger scanpath similarity to the patient group. In order to test sensitivity of scanpath similarity values discriminant analyses were performed using SPSS.

### Physical properties

To describe the physical properties of the photos that determined sensory information the following parameters were defined: luminance, luminance contrast chromaticity contrast and static contrast (Machner et al., 2012). RGB values of the photos were transferred to YCbCr color space in order to separate luminance and chromaticity. Contrasts were calculated by comparing a local patch of 2 × 2° around fixation to a global patch around fixation of the same size. For static contrasts patches were compared using black/white images of the photos which were transferred by edge detection using the canny method implemented in Matlab® with a sigma of 3 pixels.

### Statistical analyses

All statistical procedures were performed using the software package SPSS (21.0.0.1, IBM Inc, New York/USA). Analyses of variance (ANOVA) and *T*-tests were used for group comparisons on metric data level, e.g., eye movement parameters. Three-way repeated measurement analyses of variance (ANOVAs: PHOTO × GROUP × SITE) were used to test for within-subject effects, i.e., differences between photos, and for between-subject effects, i.e., group and site, in eye movement parameters. Additionally, Three-Way ANOVAs (TIME × GROUP × SITE) were performed for each photo separately whenever changes over the time course of photo presentation were of interest. There were no significant interactions of GROUP × SITE for any parameter of interest (*p* > 0.15) indicating that group differences were similar across sites. Based on these analyses, patient and controls groups were collapsed across sites. Tables with means and standard errors of all parameters of interest from each site as well as from the combined sample are available as supplementary material. Results from Two-Way ANOVAs (PHOTO × GROUP) will be reported that were followed by *post-hoc t*-tests or One-Way ANOVAs in each group separately. Greenhouse-Geisser correction of degrees of freedom was applied when repeated-measurement factors had two or more levels. Corrected *p*-values and effect sizes indicated as partial Eta (η^2^_p_) for ANOVAs and Cohen’s *d*’ for *T*-tests (Cohen, 1988) will be reported.

For group comparisons of ordinal data, e.g., subject’s emotional reaction ratings, we performed non-parametric Mann-Whitney-U tests. Spearman’s Rho was used to test for correlations between eye movement parameters and data on ordinal level, i.e., subjects’ ratings and clinical data (PANSS scores, time since first psychotic symptom, age of onset).

## Results

### General differences between photos

**Figure 2:**
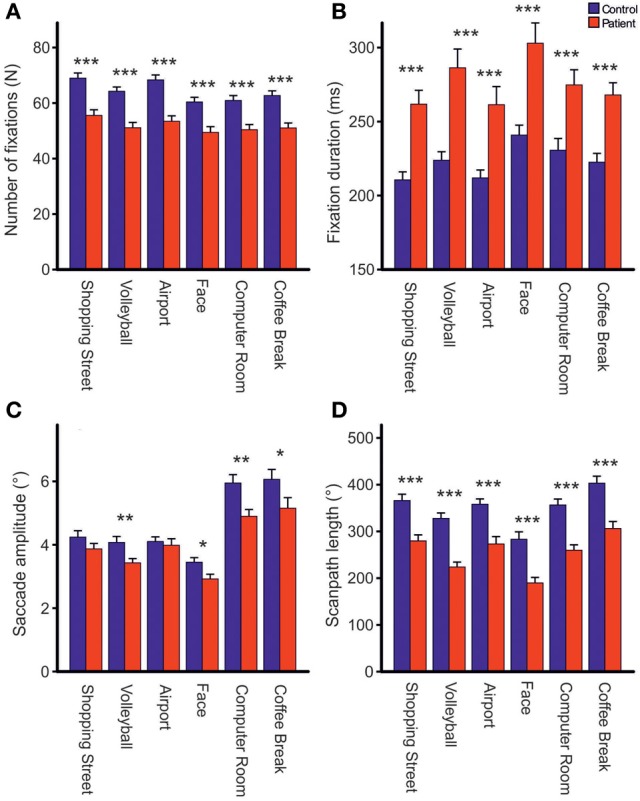
**Comparisons of the total number of fixations (A), fixation duration (B), mean saccade amplitude excluding corrective saccades >0.6° (C) and scanpath length (D) during free visual exploration of six daily life scenes between patients with schizophrenia (*N* = 32) and healthy controls (*N* = 33).** Indicated are means with standard errors, ^*^p < 0.05, ^**^p < 0.01, ^***^p < 0.001.

**Figure 3:**
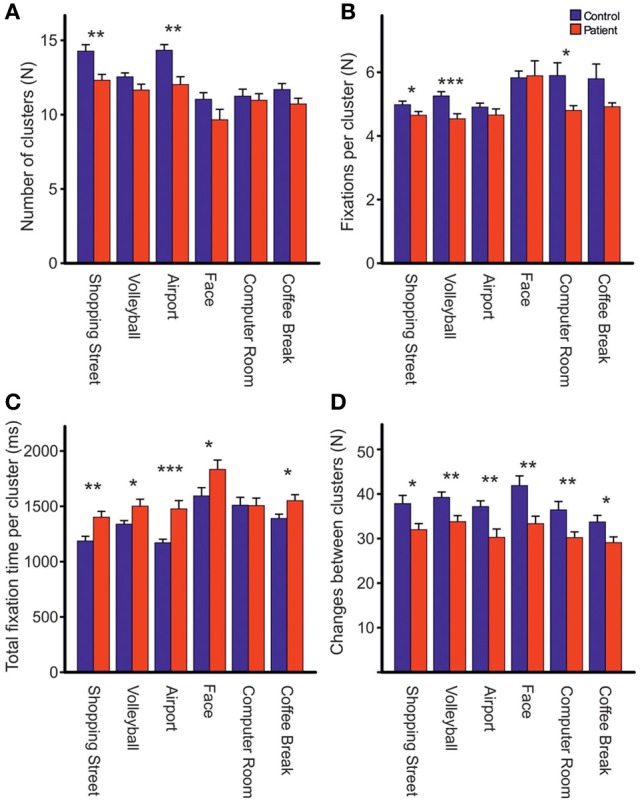
**Comparisons of the total number of clusters (A), fixations per cluster (B), total fixation time per cluster (C) and changes between clusters (D) during free visual exploration of six daily life scenes between patients with schizophrenia (*N* = 32) and healthy controls (*N* = 33).** Indicated are means with standard errors, ^*^p < 0.05, ^**^p < 0.01, ^***^p < 0.001.

Repeated measures ANOVA showed that photos differed across groups with respect to the number of fixations [*F*_photo(5, 61)_ = 15.39, *p* < 0.001, η^2^_*p*_ = 0.201], mean fixation time [*F*_photo(5, 61)_ = 9.19, *p* < 0.001, η^2^_*p*_ = 0.131], mean saccade amplitude [*F*_photo(5, 61)_ = 56.03, *p* < 0.001, η^2^_*p*_ = 0.479], and scanpath length [*F*_photo(5, 61)_ = 36.62, *p* < 0.001, η^2^_*p*_ = 0.375], Figure 2. Photo differences were also found for the parameters obtained in cluster analyses including the total number of clusters [*F*_photo(5, 62)_ = 8.97, *p* < 0.01, η^2^_*p*_ = 0.126], fixations per cluster [*F*_photo(5, 62)_ = 5.99, *p* = 0.001, η^2^_*p*_ = 0.088], total fixation time per cluster [*F*_photo(5, 57)_ = 18.54, *p* < 0.001, η^2^_*p*_ = 0.245], and changes between clusters [*F*_photo(5, 62)_ = 7.65, *p* < 0.001, η^2^_*p*_ = 0.110], Figure 3. Scanpath similarities also differed between photos [*F*_photo(5, 59)_ = 3.2, *p* = 0.014, η^2^_*p*_ = 0.051], Figure 6.

### Group differences in fixation frequency and mean fixation time

Across photos, patients made one-fifth fewer fixations per photo than controls [*patients*: 51.83 (*SD* = 8.91), *controls:* 64.25, (*SD* = 8.75), *F*_group(1, 61)_ = 31.03, *p* < 0.001, η^2^_*p*_ = 0.337, Figure 2A] and mean fixation time was longer in patients than controls [*patients*: 269 ms (*SD* = 48), *controls:* 221 ms (*SD* = 32), *F*_group(1, 61)_ = 24.23, *p* < 0.001, η^2^_*p*_ = 0.284, Figure 2B]. These group differences did not differ between photos (no interactions GROUP × PHOTO).

**Figure 4:**
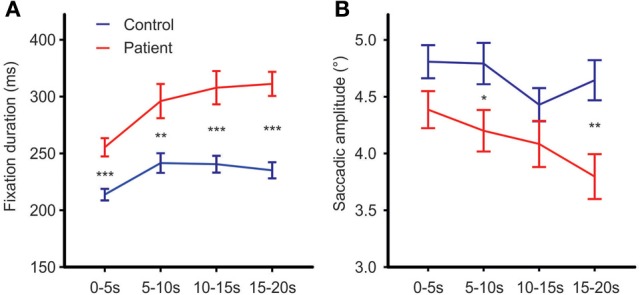
**Fixation durations (A) and mean saccadic amplitude (B) over the time course of free visual exploration of daily life scenes in patients with schizophrenia (*N* = 32) and healthy controls (*N* = 33).** The exploration time of 20 s was divided into four intervals of 5 s each. *Post-hoc t*-tests confirmed significant group differences in all four time intervals [0–5 s: *t*_(63)_ = −4.41,*p* < 0.001, *d*′ = −1.09; 5–10 s: *t*_(63)_ = −3.17, *p* = 0.002, *d*′ = −0.79; *10–15 s*: *t*_(63)_ = −4.15, *p* = 0.001, *d*′ = −1.03; 15–20 s: *t*_(63)_ = −5.99,*p* = 0.001, *d*′ = −1.49]. Indicated are means with standard errors, for *post-hoc* comparisons between groups: ^*^p < 0.05, ^**^p < 0.01, ^***^p < 0.001.

To test for changes in mean fixation time over the exploration time course, exploration time was divided into four intervals (0–5, 5–10, 10–15, and 15–20 s). Besides the expected group effect [*F*_group(1, 63)_ = 24.71, *p* < 0.001, η^2^_*p*_ = 0.282], mean fixation time differed between time intervals [*F*_time (3, 63)_ = 18.68; *p* < 0.001, η^2^_*p*_ = 0.229]. This time effect differed between groups [*F*_time × group(3, 63)_ = 2.96, *p* = 0.045, η^2^_*p*_ = 0.045, Figure 4A]. Exploring the time effect in each group separately showed significant changes in mean fixation time in both groups [*patients*: *F*_time(3, 29)_ = 10.63; *p* < 0.001, η ^2^_*p*_ = 0.255; *controls: F*_time(3, 30)_ = 10.62; *p* < 0.01, η^2^_*p*_ = 0.249]. Comparisons of regression coefficients of fixation duration on time intervals revealed that mean fixation time increase was larger in patients than controls [*t*_(63)_ = −3.6, *p* = 0.001, *d*′ = −0.89].

### Group differences in mean saccadic amplitude and scanpath length

Both groups did not differ on the number of corrective saccades (*p* = 0.072) and corrective saccades were excluded from analyses of mean saccadic amplitude. Across photos, mean saccadic amplitude in patients was smaller than in controls [*patients:* 3.90° (*SD* = 0.85), *controls:* 4.33° (*SD* = 0.77), *F*_group(1, 61)_ = 7.64,*p* < 0.01, η^2^_*p*_ = 0.0111, Figure 2C]. The scanpath length reflecting the sum of saccade amplitudes was shorter in patients than controls [*patients:* 256° (*SD* = 57), *controls:* 349° (*SD* = 59), *F*_group(1, 61)_ = 38.96, *p* < 0.001, η^2^_*p*_ = 0.390, Figure 2D]. These group differences did not differ between photos (no interactions of GROUP × PHOTO). Over the time course of exploration, mean saccadic amplitude decreased in both groups [*F*_time(3, 61)_ = 4.79, *p* < 0.01, η^2^_*p*_ = 0.073] with significant smaller amplitudes in patients compared to controls [*F*_group(1, 63)_ = 7.49, *p* < 0.01, η^2^_*p*_ = 0.106] but there was no difference in change of mean saccadic amplitude over time between groups (no interaction of TIME × GROUP), Figure 4B.

### Groups differences in cluster analyses

Across photos, patients revealed fewer fixation clusters, i.e., areas of interest, than controls [*patients:* 11.39 (*SD* = 1.72), *controls:* 12.64 (*SD* = 1.52), *F*_group(1, 62)_ = 8.97, *p* < 0.01, η^2^_*p*_ = 0.126, Figure 3A]. Patients also made fewer fixations per cluster [*patients:* 4.72 (*SD* = 0.52), *controls:* 5.21 (*SD* = 0.46), *F*_group(1, 63)_ = 16.37, *p* < 0.01, η^2^_*p*_ = 0.206, Figure 3B]. Patients had a longer total fixation time within each cluster [*patients:* 1481.19 ms (*SD* = 248.93), *controls:* 1311.07 ms (*SD* = 163.78), *F*_group(1, 63)_ = 10.66, *p* < 0.01, η^2^_*p*_ = 0.145, Figure 3C], and made fewer changes between clusters than controls [*patients:* 31.94 (*SD* = 6.61), *controls:* 37.92 (*SD* = 8.06), *F*_group (1, 62)_ = 10.69, *p* < 0.01, η^2^_*p*_ = 0.147, Figure 3D]. For all these effects, group differences did not differ between photos (no interaction PHOTO × GROUP).

### Attentional landscapes

**Figure 5:**
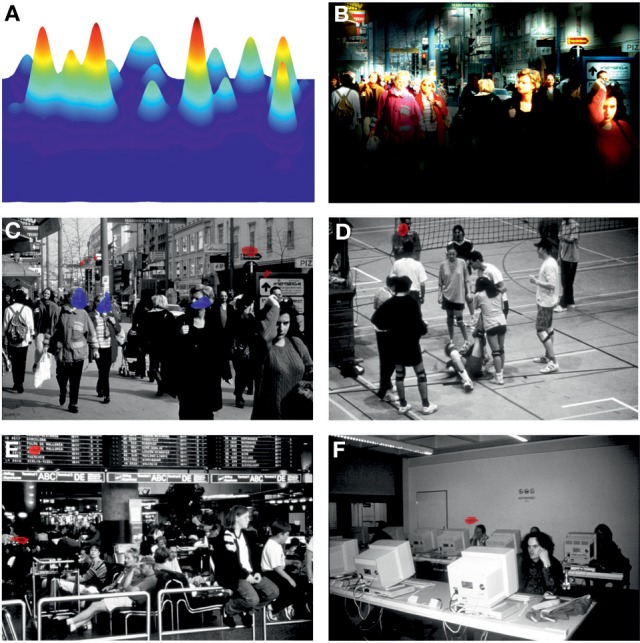
**(A) Attentional landscape of photo “Shopping Street” of a single subject.** Fixations were weighted by their duration and smoothed with a 2D Gaussian function with σ = 1° visual angle (see Materials and Methods). **(B)** Attentional landscape applied to the original photo with highlighted fields indicating areas of most intense attention allocation. **(C–F)** Results from group comparisons of attentional landscapes between patients and controls with blue areas indicating longer and red areas indicating shorter attention allocation in patients compared to controls (*p* < 0.001). For photos “Face” and “Coffee Break” attentional landscapes did not differ between groups.

Areas to which patients allocated attention differently from controls were revealed by comparisons of attentional landscapes, Figure 5. The most pronounced group difference was observed for the photo with the highest cognitive complexity score, i.e., “Shopping Street,” where patients showed significantly longer and intense attention allocation to central regions of the scene, especially faces of pedestrians, whereas controls allocated attention significantly longer to details in the periphery. While controls fixated peripheral details of the scenes “Volleyball,” “Airport,” and “Computer Room” significantly longer than patients no group differences were found related to photos “Face” and “Coffee Break.”

### Similarities between scanpaths

**Figure 6:**
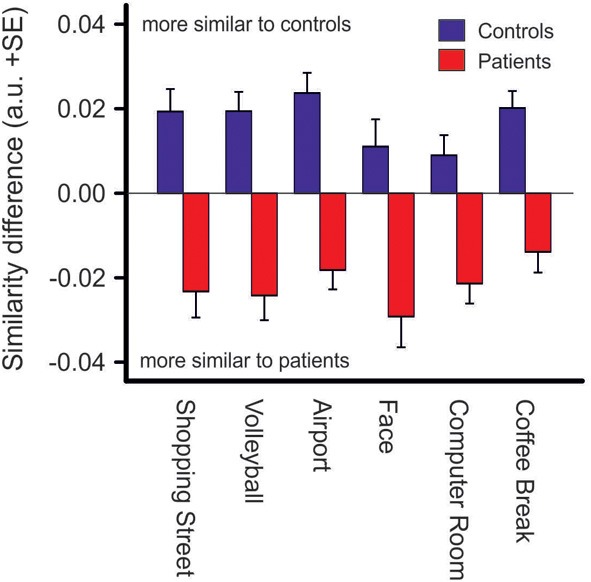
**Similarity difference scores resulting from comparisons of scanpath sequences between patients and controls.** Positive values indicate a stronger scanpath similarity to the control group whereas negative values indicate a stronger scanpath similarity to the patient group (see Materials and Methods), *p* < 0.001 for group differences in each photo.

Difference scores of scanpath similarities were quite homogenous within each group of patients and controls, Figure 6, but differed significantly between groups [*F*_group (1, 63)_ = 45.2, *p* < 0.001, η^2^_*p*_ = 0.418]. However, there was no group difference between photos (no interaction of PHOTO × GROUP). Discriminant analysis yielded an eigenvalue of 0.826, indicating that between-group variance was larger than within group-variance [Wilk’s Lambda = 0.548, χ^2^_(6)_ = 36.14, *p* < 0.001]. Group centroids were quite distinct (*patients:* −0.91, *controls:* 0.88). Eighty-five percentage of the subjects were classified correctly to their group whereas five subjects of either group were misclassified.

### Photo properties

#### Physical properties

The physical properties of fixation locations differed between photos with respect to luminance [*F*_photo(5, 62)_ = 378.83, *p* < 0.001, χ^2^_*p*_ = 0.859], luminance contrast [*F*_photo(5, 62)_ = 37.51,*p* < 0.001, η^2^_*p*_ = 0.377], chromaticity contrast [*F*_photo(5, 62)_ = 386.66, *p* < 0.001, η^2^_*p*_ = 0.862], and static contrast [*F*_photo(5, 62)_ = 97.79, *p* < 0.001, η^2^_*p*_ = 0.612] but we did not observe any group differences for any of these physical properties.

#### Cognitive complexity and emotional strain

For more detailed analyses of the effects of photo contents on visual exploration behavior, linear regression analyses of “cognitive complexity” and “emotional strain” on mean fixation time and mean saccadic amplitude were calculated for each subject using the robust-fit function within Matlab® and were then compared between groups. Regression slopes showed that in both groups mean fixation time significantly decreased with higher cognitive complexity [*patients: t*_(32)_ = −2.7, *p* < 0.01, *d*′ = 0.51, *controls*: *t*_(32)_ = −7.0, *p* < 0.001, *d*′ = 1.21] and increased with higher emotional strain [*patients: t*_(31)_ = 4.1, *p* < 0.001, *d*′ = 0.75, *controls: t*_(32)_ = 3.6, *p* < 0.01, *d*′ = 0.63]. With respect to mean saccadic amplitude, we found no relationship to cognitive complexity for neither group [*patients*: *p* = 0.13, *controls*: *p* = 0.87] but a significant decrease of mean saccadic amplitude with increasing emotional strain of the photos’ content in both groups [*patients*: *t*_(31)_ = −3.8, *p* = 0.001, *d*′ = 0.66, *controls*: *t*_(32)_ = −2.5, *p* = 0.02, *d*′ = 0.43]. In all these analyses, there were no differences between patients and controls.

#### Subjective ratings of the photos

Patients indicated having experienced more fear during exploration of the photos than controls [*Z*_(65)_ = −0.354; *p* < 0.001] but no differences between groups were found for tension, sadness, joy, aggression, for having had enough exploration time and for having grasped the content of the photo. In patients, the following correlations between eye movement parameters and subjective ratings were observed: larger saccade amplitudes were correlated with a higher rating on having grasped the photo’s content (*r* = 0.474, *p* = 0.006), and with a stronger experience of tension (*r* = 0.470, *p* = 0.007). In contrast, we did not find any correlations between emotional ratings and eye movement parameters in controls.

#### Clinical data

Relations between clinical data (Table 1) and eye movement parameters were found solely for the PANSS difference score which was correlated with mean fixation time (*r* = −0.44; *p* = 0.014) suggesting that a higher predominance of negative symptoms over positive symptoms was related to longer fixation durations.

## Discussion

The present study used an advanced approach to examine free visual exploration of social situations in patients with schizophrenia. Our results of fewer but longer fixations as well as smaller saccades resulting in shorter scanpaths in patients than controls are in line with earlier reports (Phillips and David, 1997, 1998; Williams et al., 1999; Loughland et al., 2002b; Unema et al., 2005; Bestelmeyer et al., 2006; Benson et al., 2007). Reduced fixation frequency could have nonetheless resulted in the same number of areas of interest (i.e., clusters) in patients compared to controls if patients would have made fewer fixations within each cluster. However, cluster analyses revealed that patients fixated fewer areas of interest, made fewer fixations per cluster but had a longer total fixation time within each cluster and also made fewer changes between clusters compared to controls. Following the conception of different information processing levels described by Craik and Lockhart (1972), one explanation for longer fixations in patients suggest that they were more deeply involved in semantic processing of fixated features than controls (Velichkovsky, 2002). According to this conception, preliminary processing stages are concerned with the analysis of physical or sensory features, while later stages are involved in matching the input against stored abstractions from past learning, i.e., pattern recognition and the extraction of meaning (Craik and Lockhart, 1972). Alternatively, longer fixations in patients may reflect a more general problem of disengaging attention. Attentional landscape analyses (Figure 5) illustrate those areas with longer (blue) or shorter (red) fixations in patients compared to controls. Differences between groups were most evident for the photo of the highest cognitive complexity (i.e., Shopping Street) in which patients remained longer on features in the center of the scene, whereas controls spent more time on exploring peripheral details. For three other photos, “Volleyball,” “Airport,” and “Computer Room,” we identified areas with more intense attention allocation to peripheral details in controls compared to patients.

From these results one may conclude that patients gathered less visual information from the scenes than controls. However, nearly all patients reported to have had enough time to grasp the content of the scenes suggesting that patients were not aware of their restricted visual exploration behavior. Notably, although mean fixation times were prolonged in patients, fixation durations in both groups were within the normal range of 200–350 ms (*patients:* 269 ms, *controls:* 221 ms) that has been reported in healthy individuals during free exploration. Fixation durations are determined by a range of different factors including information processing, cognitive processes or eye movement pre-programming so that fixation durations during scene exploration may vary from 100 ms to several seconds (Zingale and Kowler, 1987; Groner and Groner, 1989; Pannasch et al., 2011).

More recently, Pannasch et al. (2011) showed that during free scene exploration, fixation durations in healthy individuals are highly under direct control of stimulus information, especially when a focal-processing mode is active. This mode refers to the parafoveal attentional field in which scanning saccades with amplitudes smaller than 5° are used for more detailed information processing of a scene’s content (Velichkovsky et al., 2005). In contrast, the ambient-processing mode is accompanied by saccades larger than 5° and is thought to serve for orientation and information processing about spatial arrangements of undifferentiated visual cues (Trevarthen, 1968; Pannasch and Velichkovsky, 2009). While the focal-processing mode has been attributed to the more ventral fronto-parietal network for visual attention, the ambient-processing mode has been associated with the dorsal fronto-parietal attentional network (Corbetta et al., 2008; Pannasch et al., 2011; Marsman et al., 2012). Following this model (Pannasch and Velichkovsky, 2009; Pannasch et al., 2011), our findings of smaller mean saccadic amplitudes and longer fixation times in patients suggest that during free visual exploration of social scenes patients use a higher percentage of focal-processing than controls. Notably, saccade amplitude was correlated positively with the rating on the extent of having grasped the content of the scene in patients (but not controls), implying that those patients who used smaller saccades realized to some extent that they had missed some aspects of the scene’s content. From a brain systems perspective our findings imply that during visual exploration processing patients rely more on the ventral fronto-parietal attentional network but less on the dorsal fronto-parietal attentional network compared with controls. In schizophrenia disturbances of visual information processing along the magnocellular visual pathway and the dorsal visual stream have been related to impaired use of visual gain control and integration resulting in difficulty of modulating neural responses to take advantage of surrounding context during visual perception (Butler et al., 2008). Disturbed bottom-up visual sensorimotor information processing along the dorsal visual stream to parietal association cortex has been also reported from pursuit eye movement studies in schizophrenia using moving targets (Lencer et al., 2010, 2011). Another possible explanation for a more focal-processing mode in patients with schizophrenia comes from visual search studies that have investigated visual exploration under high attentional load, in contrast to free visual exploration used in the present study (Elahipanah et al., 2011a,b). Results from these studies imply narrower visual span size in patients, i.e., the area of the visual field from which information is extracted, especially with moving targets. This visual search dysfunction has also been related to disturbances of the dorsal visual stream (Elahipanah et al., 2011a,b).

### The when and where question

Temporal analyses showed increasing fixation durations and decreasing saccade amplitudes over the exploration time course in both groups reflecting a more “perceptive scanning” with shorter fixations in the first 5 s followed by longer fixations with allocation of attention to specific features of a scene for more “semantic/metacognitive” processing. Notably, this increase of fixation duration over time was larger in patients than controls (GROUP × TIME interaction) underlining the hypothesis that patients got more deeply involved in cognitive processing of details of the scene over the time course than controls.

Scanpath pattern analyses allow for the examination of fixation sequences, thus when and where a subject shifts attention during visual explorations. Here, analyses of scanpath similarities showed that within-group scan patterns were quite similar while scanpath patterns differed significantly between groups. Using scanpath similarities in discriminant analysis resulted in 85% of subjects being classified correctly to either the patient or the control group. Together, these findings underline the hypothesis of a specific exploration behavior in patients that differs considerably from that in controls independently of the content of a scene.

### Effects of photo properties on exploration behavior

We found clear differences of fixation, saccade and cluster parameters between photos across groups. The scene’s cognitive complexity and emotional strain were shown to have a considerable influence on scanpath patterns in both groups. In line with previous reports, fixation durations decreased with increasing complexity and fixation durations increased with increasing emotional strain while saccade amplitudes decreased with increasing emotional strain (Schrammel et al., 2009; Bradley et al., 2011). The latter observation implies a more focal-processing mode when emotional contents were explored. However, despite these obvious differences between photos, the effects of cognitive complexity and emotional strain on fixation duration and saccade amplitude did not differentiate between patients and controls (no interactions of GROUP × PHOTO in ANOVAs). This finding indicates that patients were able to adapt their visual exploration strategies to changes in cognitive complexity and emotional strain of social scenes similarly to controls. Consistent with this observation, patients also did not differ from controls in their ratings on the emotional reactions to the scenes except for anxiety which was more pronounced in patients than controls. In patients, a stronger experience of tension was correlated with larger saccade amplitudes but no further associations between emotional ratings and oculomotor parameters were observed in patients and none in controls.

Physical properties of fixation locations also did not differ between groups underlining the notion that alterations in fixation duration and saccade amplitude in patients occur independently of the stimulus’ content but rather reflect a general deficit of visual information processing as has been shown with abstract scenes or stimuli free of emotional content (Kojima et al., 2001; Bestelmeyer et al., 2006; Benson et al., 2012). Alterations in visual exploration strategies have therefore been discussed as possible biological markers for schizophrenia (Kojima et al., 2001; Bestelmeyer et al., 2006; Benson et al., 2012).

### Limitations and implications for future studies

First, we only selected six different photos of the IPT program so that stimuli material is limited. Second, although evaluations on cognitive complexity and emotional strain provided by the authors of the IPT clearly differentiated between photos they may not have been valid enough to detect differences of photo properties on exploration behavior between patients and controls. Third, despite the fact that our results of basic fixation and saccade parameters are consistent with previous reports on visual scanning behavior in chronically ill patients on long-term stable antipsychotic medication (Phillips and David, 1997, 1998; Williams et al., 1999; Loughland et al., 2002b; Bestelmeyer et al., 2006), we cannot exclude the possibility that exploration alterations in patients were influenced by medication. Follow-up studies assessing visual scanpath before and after treatment are required to evaluate the specific effect of antipsychotics on free visual exploration. Fourth, fixation durations in healthy individuals have been linked to the depth of information processing (Craik and Lockhart, 1972; Velichkovsky, 2002). However, it is difficult to determine whether increased fixation durations in patients reflect deeper information processing in patients compared to controls or whether this is due to a general slowing of processing speed (Dickinson et al., 2007) resulting in reduced gathering of visual context information. Future scanpath studies are needed that additionally compare the objective assessment of information taken from a scene between patients and controls. Fifth, special effort was undertaken to ensure similar testing conditions at both study sites, i.e., identical stimulus material and testing procedure, same eye movement recording device. However, as for most multi-site studies, there also were some differences between sites, i.e., stimulus display size was larger in Luebeck than Hamburg. Separate site specific analyses showed significant group differences for all eye movement parameters of interest with some very few exceptions (see supplementary material). In these cases, the significance threshold was not reached in either the Luebeck or the Hamburg sample probably due to lack of power in the site specific samples. In favor of this hypothesis, there was no significant interaction of GROUP × SITE for any parameter of interest combining the samples from both sites. Reducing the variance within the groups of patients and controls resulted in highly significant differences between patients and controls. We are aware of the fact that we might have ignored certain effects of stimulus display size on exploration behavior. Therefore, future studies are needed to examine the effects of stimulus display size, e.g., large vs. small visual field presentation, on free visual exploration in patients with schizophrenia.

## Conclusions

During free visual exploration in daily life situations, patients with schizophrenia seem to generally use a more focal-processing mode with longer fixations on distinct features in the center of a scene in contrast to a more ambient-processing of context information in healthy individuals. The question whether this altered visual exploration strategy in schizophrenia supports a model of how patients require more sensory evidence to inform more uncertain beliefs about the world (Friston et al., 2012) or whether the more focal-processing mode reflects a general deficit of impaired context information processing during visual perception that represents a biomarker for schizophrenia (Butler et al., 2008; Benson et al., 2012) should be subject to future studies. Despite this alteration, patients appear able to adapt their visual exploration strategies to changes in cognitive complexity, physical properties, and emotional strain similarly to healthy participants. It remains an open question whether and how cognitive remediation programs such as IPT are successful in modifying the focal-processing mode in patients into a more ambient-processing mode.

### Conflict of interest statement

The authors declare that the research was conducted in the absence of any commercial or financial relationships that could be construed as a potential conflict of interest.
