# Supplementary material for: Advanced analysis of free visual exploration patterns in schizophrenia
Source: Front Psychol. 2013 Oct 11;4:737. doi: 10.3389/fpsyg.2013.00737 (PMC3795347; doi:10.3389/fpsyg.2013.00737)
Supplement: Supplementary file 1 [file 55881_Lencer_DataSheet1.DOCX]

Supplementary material to Sprenger et al., Advanced analysis of free visual exploration patterns in schizophrenia

Below are tables providing detailed information about findings from a study of visual exploration behaviour in patients with schizophrenia and healthy participants. Eye movements were assessed while participants explored colour photos of daily life situations for 20 s. Stimuli were taken from the Integrated Psychological Training (IPT) program for patients with chronic schizophrenia (Roder et al. 1997). One part of this program focuses on remediation of social perception by improving visual exploration strategies, training patients to systematically collect visual information from a given visual scene prior to drawing conclusions and interpreting its content. Data were collected at two sites, Luebeck and Hamburg, where stimuli and assessment conditions were comparable. Statistical analysis confirmed that there were no group by site interactions.

| Data depict mean ±SE | | Combined sample | | | | Lübeck | | | | Hamburg | | | |
| --- | --- | --- | --- | --- | --- | --- | --- | --- | --- | --- | --- | --- | --- |
| Variable | Stimulus | Controls N = 33 | | Patients N = 32 | | Controls N = 16 | | Patients N = 15 | | Controls N = 17 | | Patients N = 17 | |
| Fixation frequency (N in 20s) | Average across photos | 64.3 | (1.5) | 51.8 | (1.6) | 62.1 | (2.1) | 49.3 | (2.1) | 66.3 | (2.2) | 54.1 | (2.2) |
|  | Shopping Street | 69.0 | (1.8) | 55.5 | (2.0) | 67.3 | (2.3) | 52.1 | (2.8) | 70.6 | (2.8) | 58.8 | (2.8) |
|  | Volleyball | 64.2 | (1.5) | 51.1 | (1.9) | 62.6 | (2.4) | 48.7 | (2.6) | 65.8 | (2.0) | 53.3 | (2.7) |
|  | Airport | 68.4 | (1.8) | 53.4 | (2.0) | 65.3 | (2.2) | 52.8 | (2.2) | 71.5 | (2.5) | 54.0 | (3.2) |
|  | Face | 60.4 | (1.7) | 49.4 | (2.1) | 59.2 | (2.6) | 45.9 | (2.5) | 61.5 | (2.2) | 52.6 | (3.0) |
|  | Computer Room | 60.9 | (1.7) | 50.4 | (1.8) | 57.4 | (2.3) | 47.4 | (2.6) | 64.3 | (2.4) | 53.1 | (2.5) |
|  | Coffee Break | 62.7 | (1.7) | 51.0 | (1.8) | 61.1 | (1.9) | 49.0 | (2.0) | 64.3 | (2.8) | 52.8 | (2.8) |
|  | Group effect in ANOVA | F(1,61) = | 31.03 | p < | 0.001 | F(1,29) = | 18.47 | p < | 0.001 | F(1,30) = | 14.50 | p = | 0.001 |
| Fixation duration (ms) | Average across photos | 220.6 | (5.6) | 268.6 | (8.5) | 231.1 | (7.0) | 290.8 | (12.8) | 210.7 | (8.0) | 248.9 | (9.1) |
|  | Shopping Street | 210.6 | (5.4) | 261.8 | (9.3) | 223.8 | (7.9) | 281.1 | (15.0) | 198.2 | (6.2) | 243.8 | (9.8) |
|  | Volleyball | 223.9 | (5.7) | 286.4 | (12.6) | 230.5 | (8.0) | 311.6 | (19.4) | 217.6 | (8.2) | 264.1 | (15.1) |
|  | Airport | 211.9 | (5.3) | 261.4 | (12.3) | 222.9 | (7.5) | 269.7 | (11.1) | 201.0 | (6.8) | 254.1 | (21.1) |
|  | Face | 240.9 | (6.7) | 303.0 | (16.2) | 251.5 | (10.1) | 332.3 | (29.4) | 230.9 | (8.4) | 277.2 | (14.0) |
|  | Computer Room | 230.6 | (8.0) | 274.8 | (10.2) | 247.3 | (9.7) | 295.2 | (18.0) | 214.9 | (11.4) | 256.8 | (9.3) |
|  | Coffee Break | 222.5 | (6.0) | 268.0 | (8.2) | 230.6 | (5.9) | 290.5 | (10.2) | 214.9 | (10.0) | 248.1 | (10.6) |
|  | Group effect in ANOVA | F(1,61) = | 24.23 | p < | 0.001 | F(1,29) = | 16.39 | p < | 0.001 | F(1,30) = | 11.43 | p = | 0.002 |
| Amplitude (°) | Average across photos | 4.3 | (0.1) | 3.9 | (0.2) | 4.5 | (0.2) | 4.3 | (0.2) | 4.1 | (0.2) | 3.6 | (0.2) |
|  | Shopping Street | 4.2 | (0.2) | 3.9 | (0.2) | 4.4 | (0.2) | 4.1 | (0.3) | 4.4 | (0.2) | 3.4 | (0.2) |
|  | Volleyball | 4.1 | (0.2) | 3.4 | (0.1) | 4.5 | (0.3) | 3.7 | (0.2) | 3.5 | (0.2) | 3.4 | (0.2) |
|  | Airport | 4.1 | (0.2) | 4.0 | (0.2) | 4.2 | (0.2) | 4.1 | (0.2) | 4.5 | (0.3) | 3.5 | (0.2) |
|  | Face | 3.5 | (0.1) | 2.9 | (0.2) | 3.9 | (0.2) | 3.0 | (0.2) | 3.3 | (0.2) | 2.6 | (0.2) |
|  | Computer Room | 6.0 | (0.3) | 4.9 | (0.2) | 6.0 | (0.5) | 5.9 | (0.3) | 5.1 | (0.3) | 4.7 | (0.3) |
|  | Coffee Break | 6.1 | (0.3) | 5.2 | (0.3) | 6.1 | (0.5) | 6.0 | (0.4) | 5.7 | (0.5) | 4.7 | (0.4) |
|  | Group effect in ANOVA | F(1,61) = | 7.64 | p = | 0.008 | F(1,29) = | 1.95 | p = | 0.173 | F(1,30) = | 7.45 | p = | 0.011 |

|  |  | Combined sample | | | | Lübeck | | | | Hamburg | | | |
| --- | --- | --- | --- | --- | --- | --- | --- | --- | --- | --- | --- | --- | --- |
| Variable | Stimulus | Controls N = 33 | | Patients N = 32 | | Controls N = 16 | | Patients N = 15 | | Controls N = 17 | | Patients N = 17 | |
| Scanpath similarities | Average across photos | 0.023 | (0.005) | -0.024 | (0.005) | 0.018 | (0.007) | -0.034 | (0.006) | 0.028 | (0.007) | -0.015 | (0.007) |
|  | Shopping Street | 0.019 | (0.006) | -0.027 | (0.006) | 0.013 | (0.008) | -0.037 | (0.008) | 0.024 | (0.008) | -0.019 | (0.008) |
|  | Volleyball | 0.023 | (0.004) | -0.018 | (0.006) | 0.021 | (0.006) | -0.029 | (0.007) | 0.025 | (0.006) | -0.008 | (0.009) |
|  | Airport | 0.033 | (0.006) | -0.030 | (0.006) | 0.031 | (0.009) | -0.038 | (0.008) | 0.036 | (0.008) | -0.023 | (0.009) |
|  | Face | 0.019 | (0.007) | -0.025 | (0.007) | 0.013 | (0.009) | -0.035 | (0.008) | 0.026 | (0.011) | -0.016 | (0.012) |
|  | Computer Room | 0.017 | (0.007) | -0.023 | (0.006) | 0.006 | (0.010) | -0.035 | (0.009) | 0.027 | (0.009) | -0.013 | (0.007) |
|  | Coffee Break | 0.026 | (0.007) | -0.021 | (0.007) | 0.023 | (0.008) | -0.030 | (0.009) | 0.029 | (0.010) | -0.013 | (0.011) |
|  | Group effect in ANOVA | F(1,61) = | 45.73 | p < | 0.001 | F(1,29) = | 30.12 | p < | 0.001 | F(1,30) = | 19.46 | p < | 0.001 |
| Cluster (N) | Average across photos | 12.6 | (0.3) | 11.4 | (0.3) | 12.1 | (0.4) | 10.9 | (0.4) | 13.2 | (0.4) | 11.9 | (0.5) |
|  | Shopping Street | 14.3 | (0.4) | 12.3 | (0.4) | 13.5 | (0.6) | 11.9 | (0.6) | 15.0 | (0.6) | 12.6 | (0.5) |
|  | Volleyball | 12.5 | (0.3) | 11.7 | (0.4) | 11.9 | (0.4) | 11.2 | (0.6) | 13.2 | (0.3) | 12.1 | (0.6) |
|  | Airport | 14.3 | (0.4) | 12.0 | (0.5) | 13.8 | (0.5) | 11.9 | (0.5) | 14.8 | (0.5) | 12.2 | (0.9) |
|  | Face | 11.0 | (0.4) | 9.7 | (0.7) | 10.6 | (0.7) | 9.0 | (0.6) | 11.5 | (0.6) | 10.2 | (1.2) |
|  | Computer Room | 11.2 | (0.5) | 11.0 | (0.4) | 10.9 | (0.6) | 10.8 | (0.6) | 11.6 | (0.7) | 11.1 | (0.6) |
|  | Coffee Break | 11.7 | (0.4) | 10.7 | (0.4) | 11.9 | (0.4) | 10.5 | (0.4) | 11.5 | (0.7) | 10.9 | (0.6) |
|  | Group effect in ANOVA | F(1,62) = | 10.05 | p = | 0.002 | F(1,29) = | 5.58 | p = | 0.025 | F(1,31) = | 5.18 | p = | 0.030 |
| Total fixation duration within a cluster (ms) | Average across photos | 1311 | (29) | 1481 | (44) | 1383 | (41) | 1542 | (58) | 1243 | (33) | 1428 | (64) |
|  | Shopping Street | 1187 | (42) | 1402 | (52) | 1263 | (63) | 1449 | (82) | 1115 | (53) | 1361 | (67) |
|  | Volleyball | 1339 | (32) | 1502 | (64) | 1415 | (50) | 1564 | (83) | 1268 | (32) | 1447 | (95) |
|  | Airport | 1172 | (32) | 1477 | (75) | 1227 | (45) | 1449 | (66) | 1120 | (41) | 1502 | (130) |
|  | Face | 1594 | (75) | 1834 | (84) | 1684 | (105) | 1932 | (112) | 1510 | (106) | 1748 | (123) |
|  | Computer Room | 1511 | (71) | 1507 | (68) | 1650 | (116) | 1511 | (111) | 1371 | (70) | 1503 | (86) |
|  | Coffee Break | 1391 | (39) | 1551 | 54) | 1399 | (43) | 1610 | (74) | 1381 | (67) | 1499 | (78) |
|  | Group effect in ANOVA | F(1,57) = | 8. 44 | p = | 0.005 | F(1,27) = | 2.58 | p = | 0.120 | F(1,28) = | 8.82 | p = | 0.006 |
| Number of fixations per cluster (N) | Average across photos | 5.4 | (0.1) | 4.9 | (0.1) | 5.4 | (0.1) | 4.8 | (0.1) | 5.5 | (0.2) | 5.0 | (0.2) |
|  | Shopping Street | 5.0 | (0.1) | 4.7 | (0.1) | 5.2 | (0.2) | 4.5 | (0.2) | 4.8 | (0.1) | 4.8 | (0.1) |
|  | Volleyball | 5.3 | (0.1) | 4.5 | (0.2) | 5.4 | (0.2) | 4.5 | (0.3) | 5.1 | (0.2) | 4.6 | (0.2) |
|  | Airport | 4.9 | (0.1) | 4.7 | (0.2) | 4.9 | (0.2) | 4.6 | (0.1) | 4.9 | (0.2) | 4.7 | (0.3) |
|  | Face | 5.8 | (0.2) | 5.9 | (0.5) | 6.0 | (0.3) | 5.5 | (0.4) | 5.7 | (0.3) | 6.3 | (0.8) |
|  | Computer Room | 5.9 | (0.4) | 4.8 | (0.1) | 5.5 | (0.2) | 4.6 | (0.2) | 6.3 | (0.8) | 5.0 | (0.2) |
|  | Coffee Break | 5.8 | (0.5) | 4.9 | (0.1) | 5.3 | (0.1) | 4.8 | (0.2) | 6.3 | (0.9) | 5.0 | (0.2) |
|  | Group effect in ANOVA | F(1,62) = | 8.682 | p = | 0.005 | F(1,29) = | 13.54 | p = | 0.001 | F(1,31) = | 2.27 | p = | 0.142 |

|  |  | Combined sample | | | | Lübeck | | | | Hamburg | | | |
| --- | --- | --- | --- | --- | --- | --- | --- | --- | --- | --- | --- | --- | --- |
| Variable | Stimulus | Controls N = 33 | | Patients N = 32 | | Controls N = 16 | | Patients N = 15 | | Controls N = 17 | | Patients N = 17 | |
| Changes between clusters (N) | Average across photos | 37.9 | (1.4) | 31.9 | (1.2) | 34.5 | (1.7) | 30.2 | (1.5) | 41.2 | (1.9) | 33.4 | (1.7) |
|  | Shopping Street | 37.8 | (1.8) | 32.0 | (1.4) | 34.7 | (2.3) | 31.0 | (1.6) | 40.8 | (2.7) | 32.9 | (2.1) |
|  | Volleyball | 39.2 | (1.2) | 33.8 | (1.4) | 36.0 | (1.7) | 31.7 | (1.9) | 42.3 | (1.3) | 35.6 | (1.9) |
|  | Airport | 37.2 | (1.3) | 30.3 | (1.9) | 33.8 | (1.4) | 29.1 | (2.1) | 40.4 | (1.8) | 31.3 | (3.0) |
|  | Face | 41.9 | (2.2) | 33.3 | (1.7) | 36.6 | (3.0) | 31.6 | (2.3) | 46.8 | (2.7) | 34.9 | (2.4) |
|  | Computer Room | 36.4 | (1.9) | 30.2 | (1.3) | 32.5 | (2.5) | 28.7 | (2.0) | 40.1 | (2.6) | 31.5 | (1.6) |
|  | Coffee Break | 33.7 | (1.5) | 29.1 | (1.3) | 32.8 | (1.4) | 28.5 | (1.4) | 34.7 | (2.7) | 29.6 | (2.1) |
|  | Group effect in ANOVA | F(1,62) = | 11.97 | p = | 0.001 | F(1,29) = | 3.99 | p = | 0.055 | F(1,31) = | 9.91 | p = | 0.004 |
